# Further Evaluation of Enterohemorrhagic *Escherichia coli* Gold Nanoparticle Vaccines Utilizing *Citrobacter rodentium* as the Model Organism

**DOI:** 10.3390/vaccines12050508

**Published:** 2024-05-08

**Authors:** Sarah Bowser, Angela Melton-Celsa, Itziar Chapartegui-González, Alfredo G. Torres

**Affiliations:** 1 Department of Microbiology and Immunology, The University of Texas Medical Branch, Galveston, TX 77555, USA; 2 Department of Microbiology and Immunology, Uniformed Services University of the Health Sciences, Bethesda, MD 20814, USA; 3 Department of Pathology, The University of Texas Medical Branch, Galveston, TX 77555, USA

**Keywords:** EHEC, *Citrobacter rodentium*, nanovaccines, Shiga toxin, hemolytic uremic syndrome

## Abstract

Enterohemorrhagic *E. coli* (EHEC) is a group of pathogenic bacteria that is associated with worldwide human foodborne diarrheal illnesses and the development of hemolytic uremic syndrome, a potentially deadly condition associated with Shiga toxins (Stxs). Currently, approved vaccines for human prophylaxis against infection do not exist, and one barrier preventing the successful creation of EHEC vaccines is the absence of dependable animal models, including mice, which are naturally resistant to EHEC infection and do not manifest the characteristic signs of the illness. Our lab previously developed gold nanoparticle (AuNP)-based EHEC vaccines, and assessed their efficacy using *Citrobacter rodentium*, which is the mouse pathogen counterpart of EHEC, along with an Stx2d-producing strain that leads to more consistent disease kinetics in mice, including lethality. The purpose of this study was to continue evaluating these vaccines to increase protection. Here, we demonstrated that subcutaneous immunization of mice with AuNPs linked to the EHEC antigens EscC and intimin (Eae), either alone or simultaneously, elicits functional robust systemic humoral responses. Additionally, vaccination with both antigens together showed some efficacy against Stx2d-producing *C. rodentium* while AuNP-EscC successfully limited infection with non-Stx2d-producing *C. rodentium*. Overall, the collected results indicate that our AuNP vaccines have promising potential for preventing disease with EHEC, and that evaluation of novel vaccines using an appropriate animal model, like *C. rodentium* described here, could be the key to finally developing an effective EHEC vaccine that can progress into human clinical trials.

## 1. Introduction

Diarrheagenic *Escherichia coli* is a major cause of foodborne diarrheal-disease-related morbidity and mortality, especially in children under the age of five, and even today remains an important global health concern [1]. These pathogenic bacteria can be divided into pathotypes based on distinctive characteristics, like the presence or absence of certain virulence factors, their associated clinical symptomology, and the existence of different pathogenic mechanisms [2]. One of the most common pathotypes that has led to several major outbreaks worldwide is enterohemorrhagic *E. coli* (EHEC), particularly the serotype O157:H7, which is linked to the potentially deadly condition called hemolytic uremic syndrome (HUS) due to Shiga toxin (Stx) production [2,3].

Humans usually become colonized by EHEC through the ingestion of contaminated food or water, or by contact with infected animals or animal-derived products. Once in the intestines, the bacteria use two main virulence mechanisms to cause disease [2,3]. The first is the ability to form attaching and effacing (A/E) lesions on the intestinal epithelial cells (IECs) of the colon [2]. This capability is conferred by a genomic pathogenicity island known as the locus of enterocyte effacement (LEE) that encodes for a specialized type 3 secretion system (T3SS) that injects multiple effectors into the host cell upon contact, including Tir, which translocate to the host cell membrane and binds to the bacterial outer membrane protein intimin (Eae) [2,4,5]. This interaction allows for intimate adherence of the bacteria to the IEC, triggering the subsequent reorganization of the host cell cytoskeleton and the recruitment of actin underneath the attached bacterium, forming a pedestal-like structure [2,6]. The second key virulence mechanism for EHEC is mediated by Stxs, which are exotoxins encoded within bacteriophages [2,7]. The production of Stxs is induced in the intestines; however, they can reach the bloodstream and translocate to other distant organs [2,7]. Once a Stx binds to its cellular target, mainly Gb3 receptors expressed on endothelial cells, it can be internalized and inhibit protein synthesis [7]. Secondary sequelae can develop from systemic Stxs, including HUS, which is caused by Stx-mediated damage to the kidney endothelial cells [7,8,9]. This leads to the characteristic triad of symptoms including microangiopathic hemolytic anemia, thrombocytopenia, and acute renal impairment [8,9]. 

While infections of humans with EHEC typically result in self-resolving diarrheal illness, which can be associated with symptoms like abdominal pain and hemorrhagic colitis, a portion of cases (5–10%) may advance to HUS, especially among children under 5 years old and the elderly [9]. Although the mortality rate of this condition can reach up to 10% in endemic areas, treatment options for EHEC and HUS are unfortunately limited and mostly supportive [10]. Antibiotics are contraindicated because they can induce *stx* gene expression and cause lysis of the bacterial cells, leading to an increased risk of systemic dissemination of Stxs and the further development of HUS [11,12,13]. Therefore, there has been significant effort to create a human vaccine aimed at protecting against EHEC infections and its secondary complications [14]. One major barrier in developing a successful vaccine is the absence of consistent animal models, and therefore, inefficiencies in pre-clinical testing [15]. Several animal models have been employed but all with limitations, including conventional mice, which do not exhibit the characteristic symptoms of disease after EHEC infection [15,16]. This limitation highlights the need to utilize a model that more accurately reflects EHEC-mediated human disease to fully assess vaccine efficacy. 

Our lab previously developed gold nanoparticle (AuNP)-based EHEC vaccines using antigens discovered by in silico bio- and immunoinformatic reverse vaccinology techniques [17,18,19]. Subcutaneous (s.c.) and intranasal (i.n.) immunization of BALB/c mice with AuNPs linked to two of these antigens—EscC, an LEE-encoded T3SS structural protein, and LomW, a phage-encoded outer membrane porin protein—either alone or in combination, decreased colonization of the mice by EHEC and produced vigorous systemic and mucosal antibody titers [20,21]. Although these vaccines proved useful, a complete understanding of their protective mechanisms using EHEC is challenging because BALB/c mice do not show any outward signs of disease. Therefore, we further evaluated our AuNP-based vaccines using *Citrobacter rodentium*, a pathogenic bacterium considered the murine counterpart of EHEC because it also causes A/E lesions due to the conserved LEE [22]. Furthermore, we utilized a strain of *C. rodentium* that was lysogenized with a phage carrying the *stx2d* gene (ATCC DBS770), which confers the ability to produce the toxin [23]. This strain, which encompasses both major virulence determinants of EHEC, offers a more dependable animal model and leads to consistent, measurable disease outcomes, including lethality [23].

Before assessing the EHEC AuNP vaccines, we first confirmed that we could recapitulate the established disease kinetics of both DBS770 and a non-Stx2d-producing *C. rodentium* strain (ATCC DBS771) [24]. We found that infection of female 10–12-week-old C57BL/6 mice with either strain resulted in robust colonization over 14 days and a significant increase in intestinal inflammation markers (like LCN-2), and that infection with DBS770 led to 100% mortality, significant weight loss, and inflammatory intestinal damage [24]. Next, we intranasally immunized mice using AuNPs linked to the EHEC antigens EscC, LomW, or Eae, and all three antigens stimulated both antigen- and pathogen-specific sera IgG and fecal IgA. Further in vitro functional studies demonstrated that the generated sera antibodies were bactericidal and reduced attachment of *C. rodentium* to IECs [24]. Additionally, we found that immunization with AuNP-Eae prevented death and limited intestinal damage in over 30% of DBS770-infected mice [24]. Also, both AuNP-EscC and AuNP-Eae moderately reduced the intestinal bacterial burden at 14 days post-infection (dpi) with DBS771 compared to control mice [24]. Therefore, the purpose of the current study was to use the valuable information collected from our first vaccination trial to further evaluate our EHEC AuNP vaccines. Our main goal was to test their protective efficacy by utilizing a different immunization route, a combination of antigens, and different infectious doses. We expect that by continuing to evaluate our vaccines using both Stx2d-producing and non-Stx2d-producing *C. rodentium*, we will be able to finally elucidate the correlates of protection that can be extrapolated to protect humans against EHEC.

## 2. Materials and Methods

### 2.1. Bacterial Strains and Growth Conditions

The *C. rodentium* strains utilized in this study were obtained from the American Type Culture Collection (ATCC). Stx2d-producing *C. rodentium* (ATCC DBS770) and non-Stx2d-producing *C. rodentium* (ATCC DBS771) were both routinely grown aerobically in Luria–Bertani (LB) broth at 37 °C, supplemented with antibiotics—12.5 μg/mL chloramphenicol alone (DBS770) or in combination with 25 mg/mL kanamycin (DBS771)—unless otherwise stated. The EHEC O157:H7 strain 86–24 was also normally cultured in LB broth. For animal infections, DBS770 and DBS771 were prepared as earlier outlined, with slight modifications [23,25]. The day before infection, DBS770 and DBS771 were each inoculated into LB broth supplemented with the appropriate antibiotics and statically incubated at 37 °C in 5% CO_2_ until they reached an OD_600_ of ~0.6–0.7. Cultures were centrifuged at 4000× *g* for 30 min, resuspended in 500 mL sterile 1× phosphate-buffered saline (PBS), and centrifuged once more at the same speed for 10 min. The pellets were resuspended in 60 μL of PBS (high dose, 10^9^ CFU). The high-dose stock was further diluted in PBS for the low dose (10^6^ CFU per inoculum). For in vitro assays, overnight cultures of *C. rodentium* or EHEC were diluted 1:20 in Dulbecco’s minimum essential medium (DMEM) without any supplementation and statically incubated for 4 h at 37 °C to express the T3SS components, as previously explained [4]. For in vivo experiments, bacteria in homogenized organs or feces were cultured on MacConkey agar selective media with 10 μg/mL chloramphenicol.

### 2.2. Cloning

Bacterial antigen cloning was performed as previously described [24]. Briefly, DNA from EHEC strain EDL933 was extracted with a DNeasy Blood and Tissue kit (Qiagen, Germantown, MD, USA), according to the manufacturer’s instructions. To enhance solubility of recombinant EscC (GenBank protein accession no. 12518466), the N-terminal signal sequence of the protein was predicted using the SignalP 6.0 program, and the DNA segment without the predicted signal sequence was inserted in-frame with an ^X His-tag at the C-terminus into a pET30a(+) expression vector using NdeI and XhoI (New England BioLabs, Ipswich, MA, USA). After ligation, pET30a(+)-EscC was transformed into *E. coli* DH5α-competent cells according to the manufacturer’s instructions (New England Biosciences, Ipswich, MA, USA). After confirming successful gene insertion through directional sequencing (Azenta Life Sciences, South Plainfield, NJ, USA) and gel electrophoresis, cloned plasmids were transformed into *E. coli* BL21 (DE3)-competent cells (New England BioLabs, Ipswich, MA, USA) according to the manufacturer’s instructions.

### 2.3. Protein Purification and Visualization

To induce EscC expression, overnight cultures were diluted 1:20 in 2 L of LB broth supplemented with 50 μg/mL kanamycin, grown until OD_600_ reached between 0.6 and 0.8, and induced with 1 mM of isopropyl β-D-1-thiogalactopyranoside (IPTG). Four hours after induction, bacterial cultures were centrifuged at 4000× *g* for 20 min, and the pellets were stored at −80 °C. For protein purification, thawed bacterial pellets were resuspended in 20 mL of lysis buffer (PBS supplemented with 10% glycerol, 25 mM sucrose, 1 mg/mL of lysozyme, and a tablet of cOmplete EDTA-protease inhibitor cocktail [Roche, Basel, Switzerland]). The mixture was chilled on ice for 30 min, sonicated, and subsequently pelleted at 16,000 rpm for 45 min. This was followed by multiple washes with 0.5% Sarkosyl in lysis buffer to enhance the extraction of soluble protein. Extracts containing soluble proteins were sterilized using a 0.22 µm pore-size filter and loaded onto nickel-NTA resin (Qiagen, Germantown, MD, USA) affinity columns. The protein-loaded resin was subsequently rinsed with PBS-10 mM imidazole, and EscC was eluted from the columns with PBS enriched with 10% glycerol, 25 mM sucrose, and 250 mM imidazole. The collected fractions were combined, and imidazole was removed though overnight dialysis at 4 °C in 7000 molecular weight cutoff (MWCO) Slide-A-Lyzer dialysis cassettes (Thermo Fisher Scientific, Waltham, MA, USA). Purified protein was frozen at −20 °C. Endotoxin levels were assessed using a Pierce LAL Chromogenic Endotoxin Quantification Kit (Thermo Fisher Scientific, Waltham, MA, USA) according to the manufacturer’s guidelines. Quantification of the purified protein was performed using a colorimetric bicinchoninic acid assay (BCA) following the manufacturer’s guidelines along with bovine serum albumin (BSA) standards. For protein visualization, samples were subjected to electrophoresis on SDS-PAGE gel, and protein bands were either visualized by Coomassie blue staining or transferred to a nitrocellulose membrane for Western blot analysis. The membranes were incubated overnight at 4 °C in a blocking solution consisting of 5% skim milk in PBS with 0.05% Tween-20 (PBS-T). Detection of the C-terminus 6x His-tag was assessed using a mouse anti-histidine antibody (1:5000) (Invitrogen, Carlsbad, CA, USA), followed by a horseradish peroxidase (HRP)-conjugated rabbit anti-mouse IgG secondary antibody (Southern Biotech, Birmingham, AL, USA). Protein bands were visualized by adding the ECL substrate (Thermo Fisher Scientific, Waltham, MA, USA), and the results were captured with the Amersham Imager 600 (GE Healthcare, Chicago, IL, USA). Additionally, full-length intimin (Eae) gamma protein was purified as previously described [26].

### 2.4. Coupling of Proteins onto AuNPs

The Turkevich method was used to synthesize 15 nm spherical gold nanoparticles as previously explained [27]. Briefly, heated 1 mM gold (III) chloride trihydrate solutions were reduced with 90 mM sodium citrate dihydrate. The particle size and structure were confirmed using transmission electron microscopy (TEM). To stabilize the conjugation of protein onto the AuNP surface, nanoparticles were incubated with 0.1 mM polyethylene glycol (PEG)–3400-NHS (NANOCS PG2-NSTH-3k) in water for 10 min, centrifuged at 16,000× *g* for 1 h at 4 °C, then resuspended with PBS containing the recombinant protein. For protein conjugation confirmation, the AuNPs were subjected to electrophoresis on SDS-PAGE gels, which were stained with Coomassie blue to visualize protein bands.

### 2.5. Animal Studies

Female 5-to-7-week-old C57BL/6 mice were purchased from Jackson Laboratories (Bar Harbor, ME, USA) and maintained in a biosafety level 2 (ABSL2) facility. The animals were housed in microisolator cages under pathogen-free conditions and maintained on a 12 h light cycle, with food and water available ad libitum. All animal protocols were reviewed and approved by the Institutional Animal Care and Use Committee (IACUC) of The University of Texas Medical Branch (Protocol #2112077). The mice were housed in the animal facility for at least 1 week before experimentation to allow adequate acclimation. 

### 2.6. AuNP Immunization

Female 6-to-8-week-old C57BL/6 mice were immunized subcutaneously three times at 2-week intervals with 200 μL of the vaccine formulation. The animals were administered either AuNP-EscC, AuNP-Eae, AuNP-EscC + AuNP-Eae, or unconjugated AuNPs. The vaccine formulations contained 10 μg of protein (5 μg of each protein for the combination vaccine) along with 10 μg of detoxified cholera toxin B subunit (Sigma, Cream Ridge, NJ, USA) and 500 μg Alhydrogel^®^ adjuvant 2% (InvivoGen, San Diego, CA, USA) as the adjuvants. Control mice were given unconjugated AuNPs with the same concentration of adjuvants. A total of 24 mice received each vaccine formula. For antibody titer assessment, whole blood was obtained retro-orbitally using microvette tubes without anticoagulant 1 week before the first vaccination (baseline titers) and 2 weeks after the last boost (immune titers). Sera was isolated by allowing the whole blood to clot at room temperature (RT) for 30 min, followed by centrifugation at 5000× *g* for 5 min. Sera were collected and stored at −80 °C until use. For fecal IgA titers, fecal samples were collected following the same chronology and resuspended in PBS to a final concentration of 100 mg/mL, homogenized by vortexing, and centrifuged to remove debris. Supernatants were collected and stored at −80 °C.

### 2.7. Infection, Bacterial Shedding, and Colonization

For vaccine efficacy assessment, the vaccine study, *n* = 6 mice from each vaccine group or control group were infected with either 10^9^ or 10^6^ CFU of DBS770, and 10^9^ or 10^6^ CFU of DBS771. Bacterial inocula were prepared as above, and mice were infected via feeding as previously described [25]. Briefly, a 6 μL inoculum of the bacteria was pipetted onto a ~35 mg piece of irradiated rodent chow. One piece of inoculated rodent chow was presented to 12 h fasted mice, which were monitored until the chow was fully consumed to guarantee infection. The body weight of each mouse was measured the day prior to infection, and daily thereafter. The mice were humanely euthanized upon losing 20% of their initial weight. The animals were also daily monitored for physical activity and appearance from the day before the infection until the endpoint, and individual clinical scores were assigned using the criteria in Table 1. Scores for individual parameters were combined to give the total clinical score for the day. Feces were collected before infection (day 0) and at days 2, 4, 6, and 8 post-infection to quantify bacterial shedding. Fecal samples were resuspended in 1 mL of PBS, serially diluted, and plated onto MacConkey agar supplemented with 10 μg/mL chloramphenicol for CFU enumeration. The fecal collection was stopped after 8 dpi; therefore, clinical scores beginning at 9 dpi do not include fecal observation. To evaluate bacterial colonization within the gastrointestinal tract at 14 dpi, feces, ceca, and large intestines were collected from the remaining mice. The individual fecal samples and organs were homogenized in 1 mL of sterile PBS, serially diluted, and plated onto MacConkey agar containing 10 μg/mL chloramphenicol to quantify bacterial burden through CFU enumeration. The bacterial limit of detection (LOD) was determined by the lowest dilution plated and an average of the fecal or organ weights collected.

### 2.8. Fecal LCN-2 Quantification

Fecal samples were collected from all mice prior to infection and at days 2, 4, and 6 post-infection. Samples were resuspended to a 100 mg/mL final concentration in PBS with 0.1% Tween-20, homogenized for 10 min with vortexing, and centrifuged at 14,000× *g* for 10 min. Supernatants were collected and stored at −80 °C. Concentrations of LCN-2 were determined using a DuoSet Mouse Lipocalin-2/NGAL ELISA kit (R&D Systems), according to the manufacturer’s guidelines.

### 2.9. Detection of Antigen- and Pathogen-Specific Antibodies

Serum and fecal samples were obtained from mice, and the antigen- and pathogen-specific sera IgG as well as total fecal IgA titers were assessed for individual mice by indirect enzyme-linked immunosorbent assay (ELISA). Briefly, high-binding microplates (Corning Life Sciences, Tewksbury, MA, USA) were coated with recombinant antigen [EscC or Eae (1 μg/well)] in 1× sterile PBS for the antigen-specific titers or with lysates of *C. rodentium* DBS770 or EHEC 86–24 for the pathogen-specific titers. Bacterial lysates were prepared by diluting overnight cultures 1:20 in DMEM without supplementation followed by static incubation for 4 h at 37 °C. The bacteria were pelleted and resuspended in PBS, then lysed by heat treatment (1.5 h incubation at 65 °C). A BCA assay was used to determine the protein concentration of the lysates, and the lysates were used to coat microtiter plates at a concentration of 1 μg/well. Following an overnight incubation at 4 °C, the wells were rinsed twice with washing buffer (1× PBS with 0.05% Tween-20) and blocked at RT with blocking buffer (1× PBS with 0.10% Tween-20 and 1% BSA) for 2 h. The wells were then rinsed twice with washing buffer before adding the sample diluent (1× PBS with 0.05% Tween-20 and 0.5% BSA). The sera or fecal suspensions were added to each top dilution well in duplicate, followed by 2-fold dilutions and a 2 h incubation period. The wells were then rinsed 3 times, and diluted goat anti-mouse total IgG, IgG1, IgG2c, or IgA (1:5000) (Southern Biotech, Birmingham, AL, USA [REF# 1030–05, 1071–05, 1077–05, 1040–05, respectively]) in blocking buffer was added into each well and incubated for 3 h. Plates were washed 4 times with washing buffer prior to the addition of tetramethylbenzidine (TMB) substrate solution (Invitrogen, Carlsbad, CA, USA). Stop solution (2N H_2_SO_4_) was added to each well, and the samples were immediately read at 450 and 570 nm using a microplate reader (BioTek, Paramus, NJ, USA). Endpoint titers were reported as previously described, with the reciprocal of the highest titer giving an optical density (OD) reading of at least the mean +2SD compared to the baseline sera or feces [20,21,24].

### 2.10. Serum Bactericidal Assay

Serum samples from mice immunized with AuNP-EscC, AuNP-Eae, and AuNP-EscC + AuNP-Eae (*n* = 12 from each group) were combined and either stored at −80 °C or exposed to heat inactivation (56 °C for 30 min). Serum from non-immunized C57BL/6 mice was mixed with the heat-inactivated serum (1:1) to provide an active complement source. *C. rodentium* DBS770 was prepared as outlined above and, following a 4 h incubation in DMEM, was centrifuged and resuspended in 1× PBS. Suspensions of DBS770 (1 × 10^5^ CFU) were mixed with 10% active, inactive, or inactive sera with exogenous complement in 50 µL reactions. Bacteria were incubated with serum for 1 h at 37 °C with gentle agitation. For negative controls, bacteria were exposed to serum from adjuvant-only-treated mice. After incubation, reaction mixtures were resuspended with 1× PBS and centrifuged at 4000× *g* for 15 min at RT. The PBS was removed, and the bacterial pellets were resuspended in 50 µL of fresh 1× PBS and transferred to an opaque microtiter plate (Thermo Fisher Scientific, Waltham, MA, USA). A 50 μL suspension containing 1 × 10^5^ CFU of *C. rodentium* was also transferred to the plate, as well as 50 μL of PBS. An equivalent volume of BacTiter-Glo™ Microbial Cell Viability Assay reagent (Promega, Fitchburg, WI, USA) was added to each well, and the plate was incubated for 10 min at RT with agitation. The luminescence value of each well was read on a microplate reader, and the luminescence reading of each well was corrected using the average luminescence value of 1× PBS alone. The % bacterial survival for each sample was calculated as a % of the luminescence value of the *C. rodentium* bacterial suspension. The serum bactericidal percentage was then calculated using the following formula:

$$\frac{\%\;bacterial\;survival\;in\;control\;sera\;-\;\%\;bacterial\;survival\;in\;treatment\;group\;sera}{\%\;bacterial\;survival\;in\;control\;sera}\;\times \;100$$

Each sample was run in triplicate, and the results were obtained from 4 independent experiments. 

### 2.11. Serum Adherence Inhibition Assay

C57BL/6 primary colonic epithelial cells (Cell Biologics, Chicago, IL, USA REF# C57–6047) were maintained with 5% CO_2_ in complete epithelial cell media + kit (Cell Biologics, Chicago, IL, USA REF# M6621). For adherence assays, monolayers were formed in 12-well plates by seeding them with 5 × 10^5^ cells/well in the epithelial cell media and incubating for 48 h at 37 °C with 5% CO_2_. Cultures of *C. rodentium* DBS770 were cultivated in DMEM as previously described, pelleted, then resuspended in 1× PBS. Bacteria inocula were adjusted to an MOI of 10 (5 × 10^6^ CFU [input]) and incubated for 1 h at 37 °C with gentle agitation without sera or in the presence of heat-inactivated sera (10%) from AuNP-EscC-, AuNP-Eae-, or AuNP-EscC + AuNP-Eae-immunized mice, or adjuvant-only-treated mice. Prior to infection, the primary colonic epithelial cell monolayers were washed 3 times with sterile 1× PBS. After incubation with sera, bacteria were harvested in 1 mL of fresh epithelial cell media and utilized to infect the primary colonic epithelial cell monolayers for 4 h at 37 °C with 5% CO_2_. Cells were rinsed 3 times with 1× PBS before adding 100 µL of 0.1% Triton × −100. After detachment, cell suspensions were serially diluted in PBS and cultured on LB agar plates enriched with 12.5 μg/mL chloramphenicol for CFU enumeration of attached bacteria [output]. The percentage of adhered bacteria for each condition was calculated as $\frac{CFU\;output}{CFU\;input}\times 100$. The results were obtained from 3 independent experiments using pooled sera from *n* = 12 mice.

### 2.12. Fluorescence Microscopy

For microscopy immunofluorescence analysis, infection was performed using the C57BL/6 primary epithelial cells described in the previous section. Following a 4 h infection, the cells were then washed 3 times with 1× PBS and fixed with 4% paraformaldehyde-PBS for 20 min at RT. After another 3 washes with 1× PBS, cells were stained for 1 h at RT with a solution containing PBS with tetramethyl rhodamine isothiocyanate-phalloidin (Invitrogen, Carslbad, CA, USA REF# R415) to visualize polymerized actin and DAPI (Sigma, Cream Ridge, NJ, USA REF# MBD0015) to visualize DNA (1:10,000 for both stains). The cells were washed 3 times with 1× PBS, then stained for 3 h at RT with anti-*E. coli* O plus *E. coli* K antibody coupled to fluorescein isothiocyanate (FITC) (1:1000) (Abcam, Cambridge, UK REF# AB20856) to visualize *C. rodentium*. Coverslips were mounted onto microscope slides with ProLong Gold antifade medium (Invitrogen, Carlsbad, CA, USA), visualized in an Olympus BX51 upright fluorescence microscope, and analyzed using the Image J software (Version is 1.46r). 

### 2.13. Statistical Analysis 

All statistical analysis was performed using the GraphPad Prism software (V 10.1.1). *p* values of ≤0.05 were considered statistically significant, and statistical significance was defined as follows: * *p* ≤ 0.05, ** *p* ≤ 0.01, *** *p* ≤ 0.001, **** *p* ≤ 0.0001. Quantitative data are presented as means with either the standard deviation (SD) or standard error of the mean (SEM). All data were analyzed for normality prior to conducting the corresponding statistical test. Results of the antigen-specific antibody ELISAs were analyzed using the Mann–Whitney non-parametric test. Significant differences in pathogen-specific antibody titers were determined via the Kruskal–Wallis test followed by Dunn’s multiple comparisons test. Significant differences in bacterial shedding, weight changes, clinical scores, bacterial burden in feces or organs, and serum bactericidal killing were assessed either via two-way ANOVA or mixed-effects analysis followed by Tukey’s multiple comparison test. Significant differences observed in serum adherence inhibition and colon lengths were evaluated using one-way ANOVA followed by Dunnett’s multiple comparisons test. Significance testing for comparison of survival curves was performed using the log-rank (Mantel–Cox) test. 

## 3. Results

### 3.1. Subcutaneous Immunization with AuNPs Linked to EHEC Antigens Stimulates Robust Antigen- and Pathogen-Specific Systemic Humoral Responses

**Figure 1:**
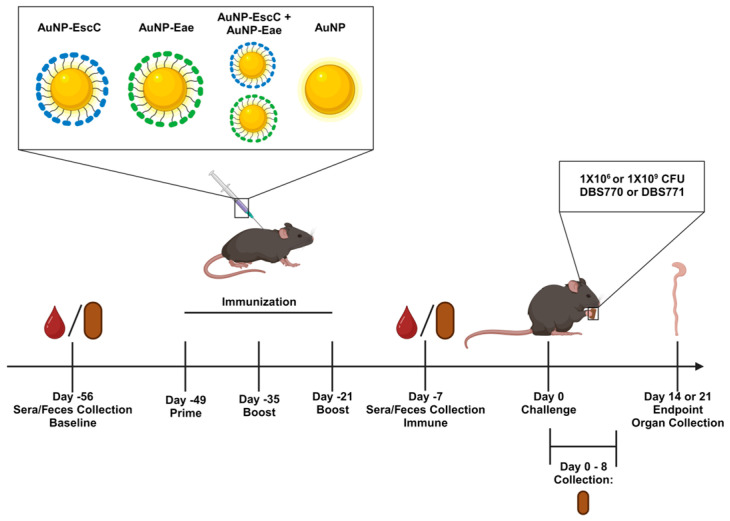
Immunization schedule. This figure illustrates the vaccination regimen of C57BL/6 mice with AuNPs conjugated to the EHEC antigens EscC, Eae, or EscC+Eae, or with unconjugated AuNPs. Immunization was followed by a feeding infection with 10^9^ CFU or 10^6^ CFU of either DBS770 or DBS771. Feces were collected during the first 8 days of infection, and both feces and organ collection at 14 or 21 dpi. Image was generated using BioRender.

Our former vaccine study using the *C. rodentium* strains utilized i.n. immunization of C57BL/6 mice with AuNPs conjugated with either EscC, LomW, or Eae, followed by infection with 10^9^ CFU of either DBS770 or DBS771 [24]. To further evaluate the effectiveness of our vaccine regimen, we next opted to subcutaneously immunize 6–8-week-old female C57BL/6 mice with vaccine formulations consisting of AuNPs linked to 10 μg of either EscC, Eae, or a combination of the two antigens (5 μg of each protein), along with adjuvants. LomW was omitted for this study due to the limited efficacy previously observed for this antigen [24]. Mice administered unconjugated AuNPs with adjuvants were used as controls. Following our well-established vaccine regimen [20,21,24], mice were immunized three times at 2-week intervals, and then infected via feeding 3 weeks after the last immunization with 10^9^ CFU (high dose) or 10^6^ CFU (low dose) of either DBS770 or DBS771 (Figure 1). The mice were examined for up to 14 or 21 days (DBS770 low dose only), and weight loss, mortality, and clinical scores were checked daily. Fecal samples were collected before infection and every day up to 8 dpi for enumeration of bacterial shedding or LCN-2 concentrations. Feces, ceca, and large intestines from DBS771-infected mice were collected at 14 dpi to enumerate bacterial burden. 

**Figure 2:**
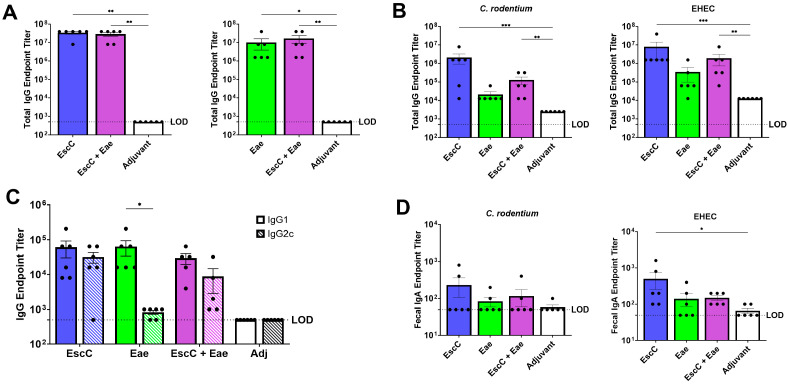
Systemic and fecal humoral responses induced by subcutaneous vaccination with AuNP vaccines. (**A**) EscC- (left) and Eae- (right) specific total serum IgG endpoint titers were quantified using plates treated with the specific antigens. (**B**) Pathogen-specific total serum IgG, (**C**) serum IgG1 and IgG2c (*C. rodentium* only), and (**D**) fecal IgA endpoint titers were quantified using plates coated with pathogen lysates. Each dot represents an individual animal, with bars showing the averages (±SEM). The dotted black line indicates the limit of detection (LOD) for each assay. Statistically significant differences in antigen-specific titers were assessed using the Mann–Whitney non-parametric test and significant differences in pathogen-specific titers (total IgG and IgA only) were conducted using the Kruskal–Wallis test followed by Dunn’s multiple comparisons test. Statistical significance in IgG1 and IgG2c titers was determined with two-way ANOVA (*, *p* ≤ 0.05; **, *p* ≤ 0.01; ***, *p* ≤ 0.001).

To assess the systemic IgG and mucosal IgA generated by the AuNP vaccines, sera and feces were obtained before the first dose and 14 days after the second boost to measure endpoint titers. All AuNP vaccines elicited significantly elevated antigen-specific serum total IgG endpoint titers compared to control animals (Figure 2A). We also found that both formulations with the EscC antigen (AuNP-EscC and AuNP-EscC+Eae) produced significantly higher total sera IgG endpoint titers specific for both *C. rodentium* and EHEC, indicating that these antibodies have cross-reactivity with both pathogens (Figure 2B). While AuNP-Eae led to increased pathogen-specific total sera IgG that were similar between *C. rodentium* and EHEC, these levels were not significant (Figure 2B). To fully understand the difference in titers with AuNP-Eae, we measured the *C. rodentium*-specific IgG sera subclasses, IgG1 and IgG2c levels. We found that while AuNP-EscC and AuNP-EscC+Eae generated similar levels of both IgG subclasses, AuNP-Eae had significantly higher IgG1 titers compared to IgG2c (Figure 2C). These titers could explain the overall lower sera total IgG seen with this antigen. Furthermore, the IgG1/IgG2c ratios imply that both AuNP-EscC and AuNP-EscC+Eae stimulate a more balanced Th2 and Th1 response because it is established that IgG1 and IgG2c, respectively, can be markers for these T cell responses in mice [28]. Lastly, we did not observe a strong pathogen-specific fecal IgA response, with only AuNP-EscC having significant titers to EHEC (Figure 2D).

### 3.2. AuNP Vaccines Elicit Serum Antibodies That Are Bactericidal and Reduce the Attachment of C. rodentium to Primary Murine Colonic Epithelial Cells

**Figure 3:**
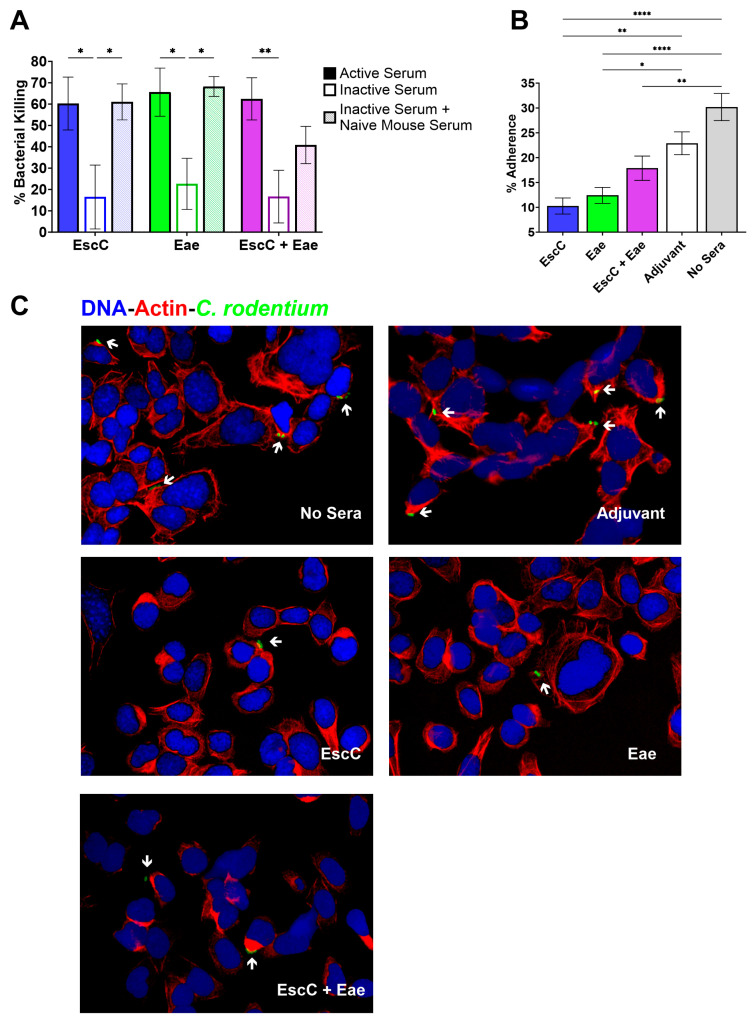
Bactericidal and adherence inhibition properties of serum antibodies elicited by the AuNP vaccines. (**A**) Bactericidal activity of the immune serum was assessed by incubating *C. rodentium* DBS770 in the presence of active, inactive, or inactive sera mixed with naïve sera from AuNP-EscC-, AuNP-Eae-, or AuNP-EscC+Eae-immunized mice for 1 h at 37 °C. Bacterial survival was calculated as a percentage of the luminescence reading of DBS770 without sera. Bacterial killing was normalized using bacteria surviving after exposure to serum from adjuvant-only-treated mice. Data are expressed as means ±SEM, obtained from combined sera collected from *n* = 12 mice. Significant differences were assessed using two-way ANOVA followed by Tukey’s multiple comparisons test. (**B**,**C**) Monolayers of primary C57BL/6 colonic epithelial cells were infected for 4 h with 5 × 10^6^ CFU of DBS770 incubated in the presence or absence (no sera) of 10% heat-inactivated sera from AuNP-EscC-, AuNP-Eae-, or AuNP-EscC+Eae-immunized mice, or from control mice, at MOI 10. Variations in bacterial binding were evaluated (**B**) quantitatively via serum adherence inhibition and (**C**) visually via fluorescence microscopy. Phalloidin rhodamine for actin (red); DAPI for nuclei (blue); anti-O/K antibody conjugated to FITC (green) for *C. rodentium*. White arrows indicate bacterial cells, and all images were taken at 60x. Significant differences were established using one-way ANOVA followed by Dunnett’s multiple comparison test (*, *p* ≤ 0.05; **, *p* ≤ 0.01; ****, *p* ≤ 0.0001).

Our lab previously demonstrated, using in vitro assays, that sera antibodies elicited by i.n. immunization of mice with our EHEC AuNP vaccines had bactericidal properties against *C. rodentium* and could limit adherence of the bacteria to a human colonic cell line [24]. Therefore, we wanted to confirm that the antibodies produced following s.c. immunization exhibited similar functionality. To elucidate the bactericidal effects of sera antibodies, we employed a high-throughput, luminescence-based assay that has been used as a convenient and consistent approach to evaluate serum bactericidal activity against several pathogens [29,30]. For this method, *C. rodentium* was mixed with either 10% active sera, heat-inactivated sera, or heat-inactivated sera supplemented with naïve mouse sera obtained from mice immunized with AuNP–protein or adjuvant-only controls. To determine bacterial viability, and therefore, the bactericidal properties of the sera, PBS-washed bacteria–sera suspensions were transferred to a microtiter plate after exposure to the sera, along with an equivalent amount of BacTiter-Glo reagent, which generates a luminescent signal proportional to the amount of ATP, and therefore, to live bacteria, present in the sample [29,30]. The luminescence reading of each sample was measured and bacterial survival was calculated as a % of the luminescence given by *C. rodentium* in PBS alone, and bacterial killing in the presence of immune sera was standardized to killing by sera from adjuvant-only-treated mice. We found significantly enhanced bacterial killing when *C. rodentium* was mixed with active serum from all three AuNP-vaccinated groups compared to bacteria exposed to heat-inactivated serum (Figure 3A). Killing was fully re-established in AuNP-EscC- and AuNP-Eae-inactivated serum when supplemented with exogenous complement, with some restoration seen in the AuNP-EscC+Eae group (Figure 3A). These findings indicate that the bactericidal effects of the elicited serum antibodies are antigen-specific and that they trigger activation of the classical complement pathway, which could be useful in host protection.

Because we have already confirmed that sera antibodies from AuNP-immunized mice can reduce the attachment of *C. rodentium* to human Caco-2 intestinal epithelial cells, we sought to use a more physiologically relevant model for this study [24]. To assess binding inhibition more fully, we instead infected monolayers of C57BL/6 primary colonic cells with *C. rodentium* that was subjected to incubation with 10% heat-inactivated serum obtained from AuNP–protein-immunized mice or control mice, or with *C. rodentium* alone (no sera). Following infection, the number of attached bacteria was calculated as a percentage of the input inoculum. Serum from AuNP-EscC- and AuNP-Eae-immunized mice successfully reduced adherence of *C. rodentium* to the primary mouse colonic cells, in contrast to bacteria incubated with serum from control mice or without serum (Figure 3B). Furthermore, serum from AuNP-EscC+Eae-immunized mice only significantly decreased adherence of the bacteria compared to the no serum group; however, it is important to note that there was no significant difference in adherence between bacteria in the presence of adjuvant-only serum and with no serum (Figure 3B). To support these quantitative results and visualize bacterial binding, monolayers were labeled with a fluorescent actin stain, DAPI (to detect DNA), and a commercially available FITC-conjugated anti-O and anti-K antibody to stain *C. rodentium*. Through fluorescence microscopy, we found that monolayers infected with *C. rodentium* that were first incubated with serum from AuNP–protein-immunized mice demonstrated less observable bacterial numbers and binding compared to cells infected with bacteria incubated with adjuvant-only-treated serum or without serum (Figure 3C). These data further confirm the ability of the serum antibodies elicited by our AuNP vaccines to block bacterial adherence to IECs, which is a crucial step in *C. rodentium* pathogenesis.

### 3.3. Immunization with AuNP-EscC+Eae Prevented Mortality in a Mouse after Infection with Stx2d-Producing C. rodentium 

**Figure 4:**
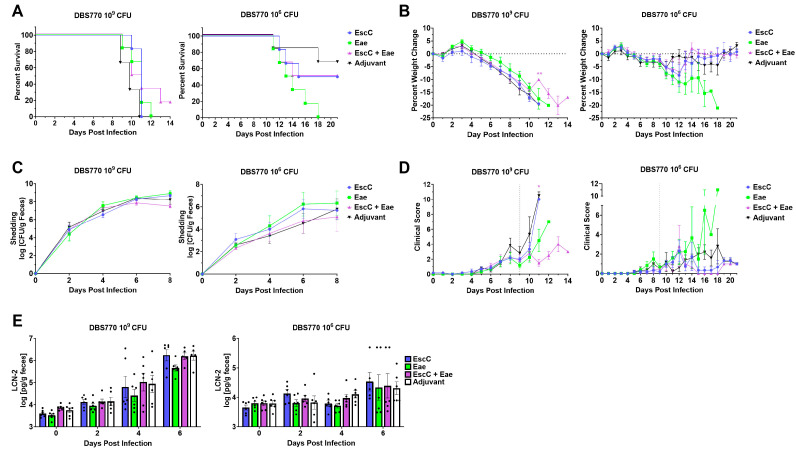
Efficacy of AuNP vaccines following infection with Stx2d-producing *C*. *rodentium* DBS770. Groups of 6 AuNP–protein-immunized or adjuvant-only-treated mice were infected with either 10^9^ or 10^6^ CFU DBS770. (**A**) The percent survival of groups was determined up to 14 dpi (10^9^ CFU) or 21 dpi (10^6^ CFU). Infected mice were monitored before (day 0) and after infection for (**B**) body weight changes, (**C**) fecal shedding of DBS770 (presented as the log CFU/g of feces), and (**D**) clinical scores, as defined in Table 1. The vertical dashed line indicates the day on which fecal criteria were no longer considered in the scores. (**E**) LCN-2 concentrations were measured in the feces of infected mice before infection (day 0) and at 2, 4, and 6 dpi, and concentrations are given as the log pg/g feces. All measurements shown are averages (±SEM) of up to 6 mice. Testing for comparison of survival curves was performed using the log-rank (Mantel–Cox) test, while significant differences in weight changes, fecal shedding, clinical scores, and fecal LCN-2 concentrations were determined by mixed-effects analysis followed by Tukey’s multiple comparison test (*, *p* ≤ 0.05; **, *p* ≤ 0.01).

Mice subcutaneously immunized with AuNP-EscC, AuNP-Eae, or AuNP-EscC+Eae, or those treated with unconjugated AuNPs (controls), were infected with either 10^9^ CFU or 10^6^ CFU of DBS770, and monitored for up to 14 or 21 dpi, respectively, to determine if the vaccines had the ability to prevent established outcomes of infection, including lethality, weight loss, fecal shedding, or intestinal inflammation. Following the high-dose infection, we found that mice in all immunization groups succumbed to infection (Figure 4A, left), lost weight (Figure 4B, left), and shed bacteria (Figure 4C, left) similarly to adjuvant-only-treated animals (AuNP unconjugated). However, one AuNP-EscC+Eae-immunized mouse (16.6%) survived and began to recover weight at 11 dpi, even until 21 dpi. Additionally, mice in all groups exhibited comparable clinical scores throughout the infection, while the individual surviving mouse never recorded scores above 4 (Figure 4D, left). Surprisingly, only AuNP-Eae-immunized mice completely succumbed to the lower-dose infection, with varying mortality seen in the other groups (Figure 4A, right), which was unexpected considering previous reports demonstrated complete lethality with this strain and method of infection at doses as low as 3.0 × 10^4^ CFU [25]. Because of this, and due to the low lethality in the control group at 14 dpi, the low-dose-infected animals were monitored until 21 dpi. During this time, we found fluctuations in weight changes (Figure 4B, right), with a steady decline only seen in the AuNP-Eae group, and no significant differences in the average shedding over 8 dpi (Figure 4C, right). We observed, however, noticeable variations in shedding by individual mice in each group, with some animals shedding as high as 10^8^ and others as low as 10^4^ on the same dpi (Figure S1B), while this phenomenon was not seen in the high-dose-infected group (Figure S1A). The clinical scores of low-dose-infected animals mirrored the other disease outcomes, with only the AuNP-Eae group having scores above 4. Lastly, fecal LCN-2 concentrations of both high- and low-dose-infected animals were measured, but there were no significant differences seen in any of the groups (Figure 4E). 

### 3.4. AuNP-EscC Limited Shedding and Intestinal Burden with Non-Stx2d-Producing C. rodentium

**Figure 5:**
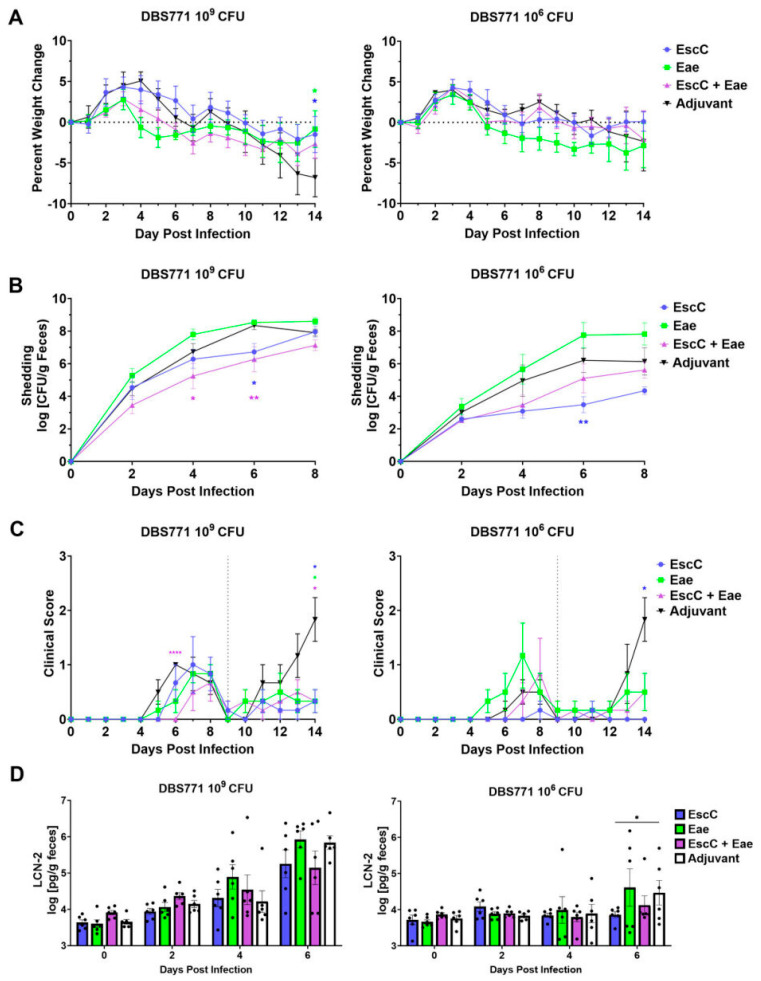
Efficacy of AuNP vaccines post-infection with non-Stx2d-producing *C. rodentium* DBS771. Groups of 6 AuNP–protein-immunized mice or control mice were infected with either 10^9^ or 10^6^ CFU DBS771. Before (day 0) and after infection, mice were monitored for (**A**) body weight changes, (**B**) fecal shedding of DBS771 (presented as log CFU/g feces), and (**C**) clinical scores, as defined in Table 1. The vertical dashed line indicates the day on which fecal criteria were no longer considered in the scores. (**D**) Fecal LCN-2 concentrations were measured before (day 0) and at 2, 4, and 6 dpi, and expressed as log pg/g feces. All depicted measurements represent the mean values (±SEM) of 6 mice, and significant differences were assessed through two-way ANOVA followed by Tukey’s multiple comparisons test (*, *p* ≤ 0.05; **, *p* ≤ 0.01; ****, *p* ≤ 0.0001).

Infection with DBS771 via the feeding method induces symptoms in mice such as fecal shedding, bacterial organ burden, and intestinal inflammation; therefore, our next step was to determine if our vaccines could limit disease manifestations caused by this strain. To compare protection in this model to the lethal strain, AuNP–protein-immunized or control (unconjugated AuNP) mice were infected via feeding with either 10^9^ CFU or 10^6^ CFU of DBS771, then monitored for up to 14 dpi. Following infection with the high dose, the weights of adjuvant-only-treated (control) mice began to decline at 9 dpi, and did not recover, and the weights of AuNP-EscC- and AuNP-Eae-immunized mice were significantly higher at 14 dpi (Figure 5A, left). Also, AuNP-EscC- and AuNP-EscC+Eae-immunized mice shed significantly less bacteria at 6 dpi, and AuNP-EscC+Eae also at 4 dpi (Figure 5B, left). Although the clinical scores of DBS771-infected mice remained relatively low compared to those of DBS770-infected mice, all the AuNP-EscC+Eae-vaccinated mice exhibited significantly lower scores at 6 dpi compared with the controls, and all the vaccinated groups at 14 dpi compared to the control group, which correlates with their decline in weight (Figure 5C, left). There were no significant differences in fecal LCN-2 concentrations among any of the groups, though there were mild decreases at 6 dpi in the AuNP-EscC and AuNP-EscC+Eae groups (Figure 5D). Furthermore, there were no differences in weight changes in the low-dose-infected groups (Figure 5A, right); however, AuNP-EscC-immunized mice shed significantly less bacteria at 6 dpi, by up to 3 log units (Figure 5B, right). Interestingly, as observed in the DBS770 low-dose-infected group, we did see variations in shedding among individual mice (Figure S1D), though shedding in AuNP-EscC-immunized mice remained consistent throughout the infection. Again, this was not seen in the DBS771 high-dose infection (Figure S1C). Finally, in accordance with the other results, the clinical scores of AuNP-EscC-immunized mice stayed at baseline for most of the infection (Figure 5C, right), with a significant reduction seen at 14 dpi compared to control mice group, and they even exhibited significantly lower fecal LCN-2 concentrations at 6 dpi (Figure 5D, right). 

**Figure 6:**
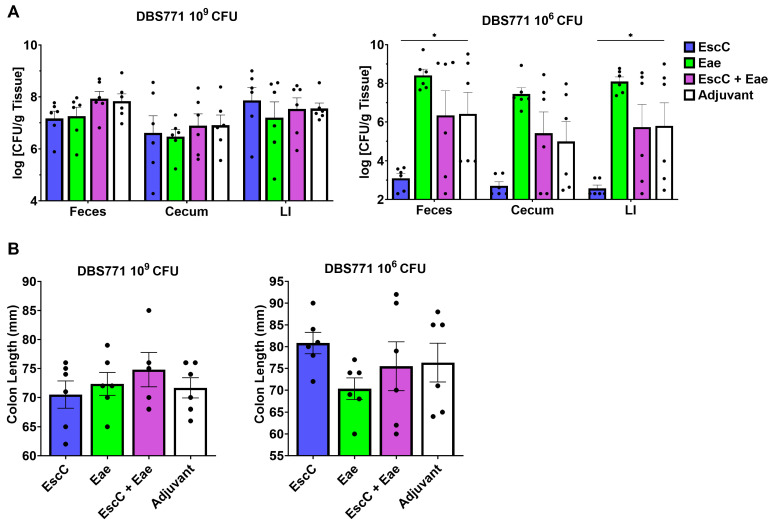
Fecal and organ analysis at 14 dpi with non-Stx2d-producing *C. rodentium* DBS771. (**A**) Feces, ceca, and large intestines (LIs) were collected from AuNP–protein-immunized or adjuvant-only-treated mice infected with either 10^9^ or 10^6^ CFU of DBS771 at 14 dpi, homogenized, and plated. Viable bacterial counts are presented as the log CFU/g tissue, and the average CFU (±SEM) of 6 animals per group is displayed. (**B**) Colons were collected from AuNP-immunized or control mice at 14 dpi. Lengths were determined by measuring the colon, beginning at the base of the cecum to the end of the distal colon. Lengths are expressed in millimeters, and the average length (±SEM) of 6 animals per group is shown. Statistical significance of bacterial burden in feces and organs was assessed by two-way ANOVA followed by Tukey’s multiple comparisons test and significant differences in colon lengths were determined by one-way ANOVA followed by Dunnett’s multiple comparisons test (*, *p* ≤ 0.05).

Next, we collected feces, ceca, and large intestines of DBS771-infected AuNP–protein-immunized and control mice at 14 dpi to evaluate differences in organ colonization. We found no significant differences at the burdens in any of the groups following the high infectious dose (Figure 6A, left); however, the AuNP-EscC low-dose-infected group exhibited significantly reduced fecal and large intestinal burdens compared to adjuvant-only-treated mice (Figure 6A, right), with a similar, although not statistically significant, trend in cecal bacterial concentrations. In fact, the bacterial burdens in all three tissues in this group were at least one log unit lower than in the fecal shedding at 8 dpi (Figure 5B, right), indicating these mice had begun to clear the infection. Additionally, we measured the colonic length, from the base of the cecum to the end of the distal colon, as this has been used before as a marker for infection and colitis development in both non-infectious and infectious models [31,32]. There were no significant differences in colonic length in any of the infected groups at any dose; however, consistent with the shedding and burden data, there was much variability in the lengths, especially of the low-dose-infected groups (Figure 6B). Of note, there were generally longer, more consistent lengths seen in the AuNP-EscC-immunized animals from low-dose infection, with one as long as 90 mm, but there was not a significant difference compared to the control group. Overall, we can conclude that the AuNP-EscC vaccine can limit infection, at least to some extent, in both a higher and lower infectious dose with non-Stx2d-producing *C. rodentium* DBS771. 

## 4. Discussion

A successful vaccine to prevent EHEC infections in humans and its secondary complications, like HUS, remains elusive, despite its considerable burden on health care. Several approaches have been used in pre-clinical testing of novel vaccines, including Stx-, attenuated bacteria-, subunit-, peptide-, plant-, DNA-, and polysaccharide-based vaccines, among others [14]. Additionally, numerous EHEC antigens have been employed with these platforms to stimulate protective immune responses, with varying efficacy [14]. Regardless of these substantial efforts, no EHEC vaccines have proceeded to human clinical trials, except for one trial that evaluated the safety of anti-Stx monoclonal antibodies in healthy individuals, but the effectiveness against infection or development of HUS was not measured [33]. A major hurdle in EHEC vaccine development is the lack of consistent tractable animal models that fully encompass EHEC-mediated disease as seen in humans [15]. Larger animals, like gnotobiotic piglets and young rabbits, have been employed, but issues like breeding, housing, and age constraints pose obvious limitations [34,35,36]. Conventional mice, the most desirable model for vaccine studies, are naturally resistant to EHEC and do not exhibit the classical signs of the illness, such as diarrhea or weight loss [16]. Microbiome-depleted mice, such as germ-free mice and those treated with streptomycin, develop morbidity and mortality following EHEC challenge, but infection in these models is Stx-mediated and does not require the LEE [37,38,39]. 

Another model that has been extensively used to study EHEC pathogenesis is *C. rodentium*, the causative agent of transmissible murine hyperplasia, because it also encodes for the LEE and triggers A/E lesions in the large intestine [22,40]. Infection of mice with wild-type *C. rodentium* can result in signs such as diarrhea, weight loss, intestinal inflammatory damage, and even death; however, the usefulness of this model in evaluating EHEC vaccines is still limited because disease severity is inconsistent among mouse strains and the pathogen does not naturally produce Stx [41,42]. To more closely mimic human infection with EHEC, Mallick, et al., lysogenized the wild-type (WT) C. rodentium strain DBS100 with a phage encoding stx2d, enabling the production of the toxin and the development of consistent disease outcomes, including death [23]. The consistency of this strain was our rationale in evaluating our previously developed EHEC AuNP vaccines using this model, along with a non-Stx2d-producing *C. rodentium* strain.

Our former study focused on intranasally immunizing C57BL/6 mice with AuNPs linked to three antigens derived from EHEC—EscC, LomW, and Eae—and then, subsequently challenging with either Stx2d-producing or non-Stx2d-producing *C. rodentium* strains to evaluate vaccine efficacy [24]. We demonstrated that while these antigens could stimulate functional, antigen- and pathogen-specific antibodies, the protection conferred by our vaccines was modest. Therefore, the goal of the current work was to further evaluate our AuNPs by modifying the vaccine regimen to enhance its effectiveness. C57BL/6 mice were instead subcutaneously immunized to boost systemic immune responses, with Alhydrogel^®^, a recognized Th2 inducer, added as an adjuvant [43]. Our hope was to increase serum IgG since this antibody isotype is known to be protective against *C. rodentium* [44]. This contrasts with EHEC, with IgA in the gut being indispensable in defense against this pathogen, so the known mucosal adjuvant cholera toxin B subunit was included in the formulation as well [45,46,47]. Additionally, only the antigens EscC and Eae were used, either alone or in combination, considering they previously exhibited the best protection, and because the EHEC proteins share a high identity with those encoded by the LEE of *C. rodentium* (96% and 78% identity, respectively) [48]. Moreover, Eae has been utilized extensively in pre-clinical vaccine studies, showing varying efficacy against both *C. rodentium* and EHEC [14,49]. Subcutaneous immunization with AuNPs linked to these antigens, either alone or in combination, induced almost 10,000 times higher antigen-specific total serum IgG endpoint titers (Figure 2A) compared to i.n. immunization [24]. *C. rodentium*-specific serum IgG1 and IgG2c titers were also measured, and both AuNP-EscC and AuNP-EscC+Eae generated similar titers of both subtypes, while AuNP-Eae immunization caused skewing towards IgG1 (Figure 2C), which could explain the lower total serum IgG (Figure 2B). Further investigation into the cell-mediated responses following vaccination is warranted to explore these differences. Nonetheless, sera antibodies from all vaccine groups displayed bactericidal capabilities against *C. rodentium*, and reduced adherence of the bacteria to murine primary colonic cells, which was confirmed quantitatively and visually through fluorescence microscopy (Figure 3). Regarding mucosal responses, insignificant pathogen-specific fecal IgA was observed following subcutaneous immunization, except for EscC against EHEC (Figure 2D), which is due to the route of vaccination, suggesting that a combination of routes and/or other adjuvants may be needed to balance systemic and mucosal responses. After in vitro antibody characterization, the subsequent step was to evaluate whether these immune responses would efficiently guard against infection from both Stx2d-producing and non-Stx2d-producing *C. rodentium* at different infectious doses. 

It has been reported that the infectious dose of EHEC necessary to infect humans can be as low as 10–100 CFU [50]. In mice, however, much higher doses (10^8^–10^9^ CFU) are typically used to instigate colonization, in accordance with what is used in WT *C. rodentium* infections [16]. Following the creation of Stx2d-producing *C. rodentium*, Flowers, et al., demonstrated that much lower inocula, i.e., 3 × 10^4^ CFU, given via the feeding method can still lead to consistent disease outcomes and mortality [25]. Because of this, AuNP–protein-immunized mice were challenged with both a high (10^9^ CFU) and low dose (10^6^ CFU) of either DBS770 (Stx2d-producing) or DBS771 (non-Stx2d-producing). Mice in all vaccine groups infected with the high dose of DBS770 lost weight and succumbed to infection similarly to the control group (Figure 4A, left), except for one AuNP-EscC+Eae-immunized animal that survived. It is important to note that colonization of the mice, measured via fecal shedding of the bacteria, was consistent among animals in all groups during the infection (Figure 4C, right; Figure S1A). This contrasts with what was seen in mice infected with the low dose of DBS770, with variations in mortality (Figure 4A, right), weight fluctuations (Figure 4B, right), and shedding (Figure 4C, right; Figure S1B) observed among individual mice, even in the control group. This occurrence was unexpected considering the results obtained from Flowers, et al. We do not expect that this is due to a delay in colonization because the mice began to recover. Explanations could be the age at which the mice were infected in our study (12–14 weeks old) compared to the past report (6–8 weeks old) or variations in intestinal microbiota composition, which have been proven to affect resistance to C. rodentium infection [51]. Furthermore, the susceptibility of AuNP-Eae-immunized mice could be due to the Th2-polarized response to this antigen, leading to an impaired Th1 response, which has been shown to aid in clearance of *C. rodentium* [52]. Subsequent studies will focus on understanding these differences in colonization and immune responses, as well as tailoring the vaccine regimen and infectious dose to obtain a balance of protective immunity and consistent disease kinetics. 

The non-lethal DBS771 strain was also used to measure AuNP–protein vaccine efficacy. Infection with the high dose in subcutaneously immunized mice resulted in some protection against weight loss at 14 dpi (Figure 5A, left), reduced fecal shedding at 4 and 6 dpi (Figure 5B, left), and lower clinical scores at 6 and 14 dpi (Figure 5C, left). However, we did not find any significant differences in fecal or organ burdens after 2 weeks of infection (Figure 6A, left). Importantly, the shedding by individual mice within each group was similar by 8 dpi (Figure S1C), with high variations also seen following infection with the low dose of this strain (Figure S1D). Nonetheless, AuNP-EscC-immunized mice maintained lower average shedding concentrations (Figure 5B, right), with a significant reduction compared to the control group at 6 dpi. These findings correlated with consistently low clinical scores (Figure 5C, right), a significant reduction in fecal LCN-2 concentrations (Figure 5D, right), and reduced fecal and organ burdens at 14 dpi (Figure 6A, right) in this group. These results imply that regardless of the differences in colonization progression seen in the other low-dose-challenged animals, AuNP-EscC successfully limited infection with DBS771. Moreover, this could indicate that to see the same protection against Stx2d-producing *C. rodentium*, and therefore EHEC, immunity against the toxin itself might be necessary.

## 5. Conclusions

In summary, we confirmed that s.c. immunization with our AuNP-conjugated vaccines induced substantial functional systemic immune responses and that both St2d-producing and non-Stx2d-producing *C. rodentium* offer viable models to assess their efficacy, both for colonization and lethality. We continued to validate the disease kinetics of either strain using both high and low doses, with the latter demonstrating high variability in the severity of disease in the mice. Additionally, we showed that EscC alone could limit infection with either dose of the non-Stx2d-producing strain. Overall, the outcomes provided by our study will help to inform the scientific community about future modifications required to the vaccine regimen, as defined herein, to maximize the protective efficacy of the EHEC AuNP vaccines. The results of our vaccine studies suggest that both the antigens used, and the route of inoculation are relevant in generating systemic immune responses. Hence, we hypothesize that a combination of immunization routes with these vaccine formulations could generate effective protection.

## Figures and Tables

**Table 1:** Criteria used for clinical scores.

Parameter	Description	Score
Appearance	Normal (smooth coat, eyes wide open and round) Fur: dulled and ruffled; Eyes: wide open and round Fur: dulled and ruffled; Eyes: almond-shaped lid Hunched; Fur: dulled and ruffled; Eyes: slit-like lid	0 1 2 3
Activity	Normal (fast running and active) Decreased mobility Wobbly/weak Inactive	0 1 2 3
Respiratory Rate	Normal breathing Increased breathing (rapid, shallow, double normal rate) Abdominal breathing (open-mouth breathing/gasping)	0 1 2
Weight Loss	<5% weight loss or weight gain 5–10% weight loss 10–20% weight loss >20% weight loss	0 1 2 3
Fecal	Normal fecal production Softer fecal pellets; takes longer than 10 min to produce Some diarrhea; takes longer than 10 min to produce Only diarrhea; does not produce	0 1 2 3

## Data Availability

The data presented in this study are available on request from the corresponding authors.

## Supplementary Materials

The following supporting information can be downloaded at: https://www.mdpi.com/article/10.3390/vaccines12050508/s1, Figure S1: Shedding of DBS770 and DBS771 in infected animals at 2, 4, 6, and 8 dpi.

## References

[1] WHO Diarrhoeal Disease. https://www.who.int/news-room/fact-sheets/detail/diarrhoeal-disease.

[2] Kaper J.B., Nataro J.P., Mobley H.L.T. (2004). Pathogenic *Escherichia coli*. Nat. Rev. Microbiol..

[3] CDC *E. coli* (*Escherichia coli*). https://www.cdc.gov/ecoli/index.html.

[4] Elliott S.J., Sperandio V., Giron J.A., Shin S., Mellies J.L., Wainwright L., Hutcheson S.W., McDaniel T.K., Kaper J.B. (2000). The locus of enterocyte effacement (LEE)-encoded regulator controls expression of both LEE- and non-LEE-encoded virulence factors in Enteropathogenic and Enterohemorrhagic *Escherichia coli*. Infect. Immun..

[5] Franzin F.M., Sircili M.P. (2015). Locus of enterocyte effacement: A pathogenicity island involved in the virulence of Enteropathogenic and Enterohemorrhagic *Escherichia coli* subjected to a complex network of gene regulation. BioMed Res. Int..

[6] Stevens M.P., Frankel G.M. (2014). The Locus of Enterocyte Effacement and Associated Virulence Factors of Enterohemorrhagic *Escherichia coli*. Microbiol. Spectr..

[7] Melton-Celsa A.R. (2014). Shiga Toxin (Stx) Classification, Structure, and Function. Microbiol. Spectr..

[8] Joseph A., Cointe A., Kurkdjian P.M., Rafat C., Hertig A. (2020). Shiga Toxin-Associated Hemolytic Uremic Syndrome: A Narrative Review. Toxins.

[9] Mallinckrodt E., Tarr P.I., Gordon C.A., Chandler W.L. (2005). Shiga-toxin-producing *Escherichia coli* and haemolytic uraemic syndrome. Lancet.

[10] Spinale J.M., Ruebner R.L., Copelovitch L., Kaplan B.S. (2013). Long-term outcomes of Shiga toxin hemolytic uremic syndrome. Pediatr. Nephrol..

[11] Freedman S.B., Xie J., Neufeld M.S., Hamilton W.L., Hartling L., Tarr P.I., Nettel-Aguirre A., Chuck A., Lee B., Johnson D. (2016). Shiga Toxin–Producing *Escherichia coli* Infection, Antibiotics, and Risk of Developing Hemolytic Uremic Syndrome: A Meta-analysis. Clin. Infect. Dis..

[12] Wong C.S., Jelacic S., Habeen R.L., Watkins S.L., Tarr P.I. (2000). The Risk of Hemolytic-Uremic Syndrome after Antibiotic Treatment of *Escherichia coli* O157:H7 Infections. N. Engl. J. Med..

[13] Kakoullis L., Papachristodoulou E., Chra P., Panos G. (2019). Shiga toxin-induced haemolytic uraemic syndrome and the role of antibiotics: A global overview. J. Infect..

[14] Rojas-Lopez M., Monterio R., Pizza M., Desvaux M., Rosini R. (2018). Intestinal pathogenic *Escherichia coli*: Insights for vaccine development. Front. Microbiol..

[15] Ritchie J.M. (2014). Animal Models of Enterohemorrhagic *Escherichia coli* Infection. Microbiol. Spectr..

[16] O’Brien A.D., Mohawk K.L. (2011). Mouse models of *Escherichia coli* O157:H7 Infection and Shiga Toxin Injection. J. Biomed. Biotechnol..

[17] García-Angulo V.A., Kalita A., Kalita M., Lozano L., Torres A.G. (2014). Comparative genomics and immunoinformatics approach for the identification of vaccine candidates for Enterohemorrhagic *Escherichia coli* O157:H7. Infect. Immun..

[18] Tapia D., Ross B.N., Kalita A., Kalita M., Hatcher C.L., Muruato L.A., Torres A.G. (2016). From in silico protein epitope density prediction to testing *Escherichia coli* O157: H7 vaccine candidates in a murine model of colonization. Front. Cell. Infect. Microbiol..

[19] Kalita A., Kalita M., Torres A.G. (2014). Exploiting the power of OMICS approaches to produce *E. coli* O157 vaccines. Gut Microbes.

[20] Sanchez-Villamil J.I., Tapia D., Torres A.G. (2019). Development of a gold nanoparticle vaccine against Enterohemorrhagic *Escherichia coli* O157:H7. mBio.

[21] Sanchez-Villamil J.I., Tapia D., Torres A.G. (2022). Optimization of Multivalent Gold Nanoparticle Vaccines Eliciting Humoral and Cellular Immunity in an In Vivo Model of Enterohemorrhagic *Escherichia coli* O157:H7 Colonization. mSphere.

[22] Collins J.W., Keeney K.M., Crepin V.F., Rathinam V.A., Fitzgerald K.A., Finlay B.B., Frankel G. (2014). Citrobacter rodentium: Infection, inflammation and the microbiota. Nat. Rev. Microbiol..

[23] Mallick E.M., McBee M.E., Vanguri V.K., Melton-Celsa A.R., Schlieper K., Karalius B.J., O’brien A.D., Butterton J.R., Leong J.M., Schauer D.B. (2012). A novel murine infection model for Shiga toxin–producing *Escherichia coli*. J. Clin. Investig..

[24] Bowser S., Melton-Celsa A., Chapartegui-González I., Torres A.G. (2024). Efficacy of EHEC gold nanoparticle vaccines evaluated with the Shiga toxin-producing Citrobacter rodentium mouse model. Microbiol. Spectr..

[25] Flowers L.J., Bou Ghanem E.N., Leong J.M. (2016). Synchronous disease kinetics in a murine model for Enterohemorrhagic *E. coli* infection using food-borne inoculation. Front. Cell. Infect. Microbiol..

[26] Gansheroff L.J., Wachtel M.R., O’Brien A.D. (1999). Decreased adherence of Enterohemorrhagic *Escherichia coli* to HEp-2 cells in the presence of antibodies that recognize the C-terminal region of intimin. Infect. Immun..

[27] John Turkevich B., Cooper Stevenson P., Hillier J. (1951). A Study of the Nucleation and Growth Processes in the Synthesis of Colloidal Gold. Discuss. Faraday Soc..

[28] Stevens T.L., Bossie A., Sanders V.M., Fernandez-Botran R., Coffman R.L., Mosmann T.R., Vitetta E.S. (1988). Regulation of antibody isotype secretion by subsets of antigen-specific helper T cells. Nature.

[29] Clow F., O’Hanlon C.J., Christodoulides M., Radcliff F.J. (2019). Feasibility of Using a Luminescence-Based Method to Determine Serum Bactericidal Activity against Neisseria gonorrhoeae. Vaccines.

[30] Aruta M.G., Carducci M., Micoli F., Necchi F., Rossi O. (2021). Increasing the High Throughput of a Luminescence-Based Serum Bactericidal Assay (L-SBA). BioTech.

[31] Chassaing B., Aitken J.D., Malleshappa M., Vijay-Kumar M. (2014). Dextran Sulfate Sodium (DSS)-Induced Colitis in Mice. Curr. Protoc. Immunol..

[32] Bhinder G., Sham H.P., Chan J.M., Morampudi V., Jacobson K., Vallance B.A. (2013). The Citrobacter rodentium Mouse Model: Studying Pathogen and Host Contributions to Infectious Colitis. J. Vis. Exp..

[33] López E.L., Contrini M.M., Glatstein E., González Ayala S., Santoro R., Allende D., Ezcurra G., Teplitz E., Koyama T., Matsumoto Y. (2010). Safety and pharmacokinetics of urtoxazumab, a humanized monoclonal antibody, against Shiga-like toxin 2 in healthy adults and in pediatric patients infected with Shiga-like toxin-producing *Escherichia coli*. Antimicrob. Agents Chemother..

[34] Francis D.H., Collins J.E., Duimstra J.R. (1986). Infection of gnotobiotic pigs with an *Escherichia coli* O157:H7 strain associated with an outbreak of hemorrhagic colitis. Infect. Immun..

[35] Tzipori S., Wachsmuth I.K., Chapman C., Birner R., Brittingham J., Jackson C., Hogg J. (1986). The Pathogenesis of Hemorrhagic Colitis Caused by *Escherichia coli* O157:H7 in Gnotobiotic Piglets. J. Infect. Dis..

[36] Potter M.E., Kaufmann A.F., Thomason B.M., Blake P.A., Farmer J.J. (1985). Diarrhea Due to *Escherichia coli* O157:H7 in the Infant Rabbit. J. Infect. Dis..

[37] Wadolkowski E.A., Sung L.M., Burris J.A., Samuel J.E., O’Brien A.D. (1990). Acute renal tubular necrosis and death of mice orally infected with *Escherichia coli* strains that produce Shiga-like toxin type II. Infect. Immun..

[38] Wadolkowski E.A., Burris J.A., O’Brien A.D. (1990). Mouse model for colonization and disease caused by Enterohemorrhagic *Escherichia coli* O157:H7. Infect. Immun..

[39] Eaton K.A., Friedman D.I., Francis G.J., Tyler J.S., Young V.B., Haeger J., Abu-Ali G., Whittam T.S. (2008). Pathogenesis of renal disease due to enterohemorrhagic *Escherichia coli* in germ-free mice. Infect. Immun..

[40] Rahman T., Seraj M.d.F., Islam M.d.M., Rahman T., Seraj M.d.F., Islam M.d.M. (2018). Citrobacter rodentium, a Gut Pathogen: The Yin and the Yang of Its Pathophysiology, Immunity and Clinical Manifestation in Mice. Adv. Microbiol..

[41] Mundy R., MacDonald T.T., Dougan G., Frankel G., Wiles S. (2005). Citrobacter rodentium of mice and man. Cell. Microbiol..

[42] Carson D., Barry R., Hopkins E.G., Roumeliotis T.I., García-Weber D., Mullineaux-Sanders C., Elinav E., Arrieumerlou C., Choudhary J.S., Frankel G. (2020). *Citrobacter rodentium* induces rapid and unique metabolic and inflammatory responses in mice suffering from severe disease. Cell. Microbiol..

[43] Hogenesch H. (2012). Mechanism of immunopotentiation and safety of aluminum adjuvants. Front. Immunol..

[44] Maaser C., Housley M.P., Iimura M., Smith J.R., Vallance B.A., Finlay B.B., Schreiber J.R., Varki N.M., Kagnoff M.F., Eckmann L. (2004). Clearance of Citrobacter rodentium requires B cells but not secretory immunoglobulin A (IgA) or IgM antibodies. Infect. Immun..

[45] Holmgren J., Lycke N., Czerkinsky C. (1993). Cholera toxin and cholera B subunit as oral—Mucosal adjuvant and antigen vector systems. Vaccine.

[46] Nagano K., Taguchi K., Tokoro S., Tatsuno I., Moria H. (2014). Adhesion of Enterohemorrhagic *Escherichia coli* O157:H7 to the Intestinal Epithelia Is Essential for Inducing Secretory IgA Antibody Production in the Intestine of Mice. Biol. Pharm. Bull..

[47] Ma Z., Zhang H., Shang W., Zhu F., Han W., Zhao X., Han D., Wang P.G., Chen M. (2014). Glycoconjugate Vaccine Containing *Escherichia coli* O157:H7 O-Antigen Linked with Maltose-Binding Protein Elicits Humoral and Cellular Responses. PLoS ONE.

[48] Deng W., Li Y., Vallance B.A., Finlay B.B. (2001). Locus of enterocyte effacement from Citrobacter rodentium: Sequence analysis and evidence for horizontal transfer among attaching and effacing pathogens. Infect. Immun..

[49] Ghaem-Maghami M., Simmons C.P., Daniell S., Pizza M., Lewis D., Frankel G., Dougan G. (2001). Intimin-specific immune responses prevent bacterial colonization by the attaching-effacing pathogen Citrobacter rodentium. Infect. Immun..

[50] Rahal E.A., Kazzi N., Nassar F.J., Matar G.M. (2012). *Escherichia coli* O157:H7-Clinical aspects and novel treatment approaches. Front. Cell. Infect. Microbiol..

[51] Osbelt L., Thiemann S., Smit N., Lesker T.R., Schröter M., Gálvez E.J.C., Schmidt-Hohagen K., Pils M.C., Mühlen S., Dersch P. (2020). Variations in microbiota composition of laboratory mice influence Citrobacter rodentium infection via variable short-chain fatty acid production. PLoS Pathog..

[52] Silberger D.J., Zindl C.L., Weaver C.T. (2017). Citrobacter rodentium: A model enteropathogen for understanding the interplay of innate and adaptive components of type 3 immunity. Mucosal Immunol..

