# Supplementary figures and images for: Further Evaluation of Enterohemorrhagic Escherichia coli Gold Nanoparticle Vaccines Utilizing Citrobacter rodentium as the Model Organism
Source: Vaccines (Basel). 2024 May 8;12(5):508. doi: 10.3390/vaccines12050508 (PMC11125983; doi:10.3390/vaccines12050508)

**Figure S1.** Shedding of DBS770 and DBS771 in infected animals at 2, 4, 6, and 8 dpi.

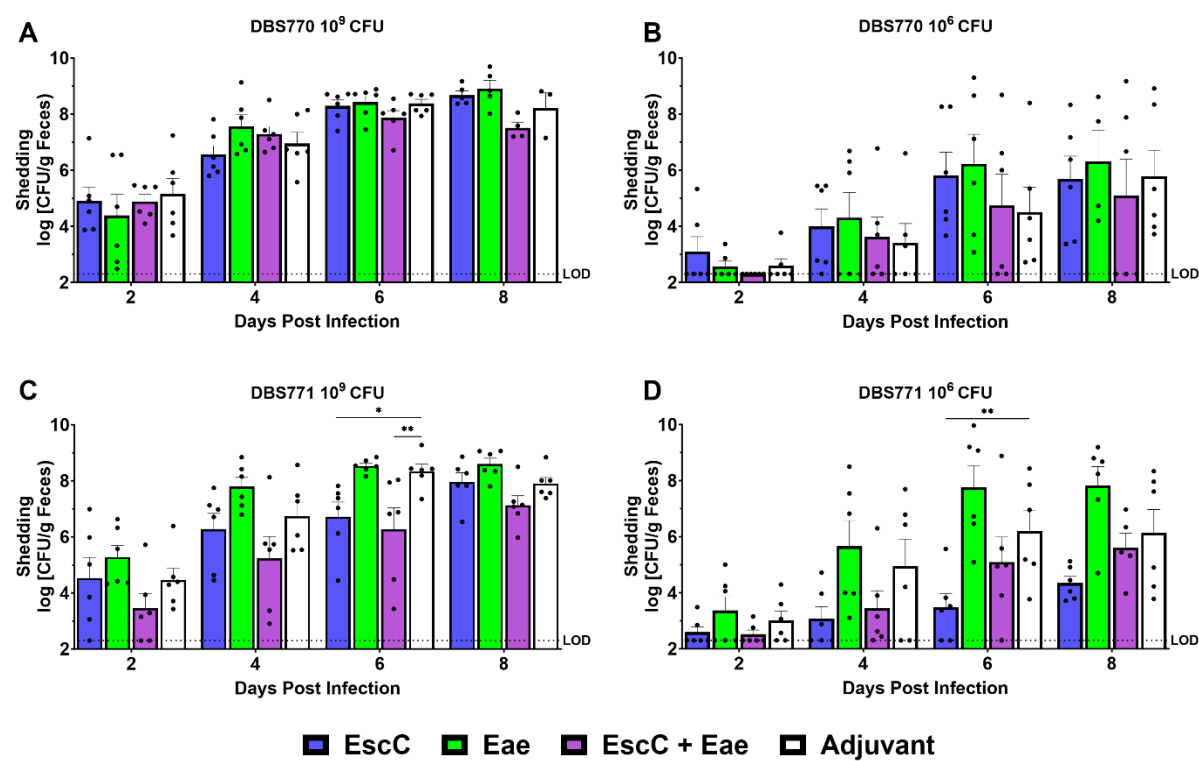

Supplement: Supplementary file 1 [file vaccines-12-00508-s001.zip › vaccines-2953580-supplementary.pdf]
